# SEELE: Sense-Driven Edge-Cloud Foreground–Background Split Rendering for Immersive Media Services

**DOI:** 10.3390/s26175561

**Published:** 2026-09-01

**Authors:** Yuxuan Xiao, Han Xiao, Chuxing Fang, Shaoyun Wu, Mingyu Zhao, Enbo Wang, Changqiao Xu

**Affiliations:** 1 State Key Laboratory of Networking and Switching Technology, Beijing University of Posts and Telecommunications, Beijing 100876, China; winderings@bupt.edu.cn (Y.X.); fcxfcx@bupt.edu.cn (C.F.); cqxu@bupt.edu.cn (C.X.); 2 Huawei Technologies Company Limited, Shanghai 201799, China; wushaoyun@huawei.com (S.W.); zhaomingyu2@huawei.com (M.Z.); wangenbo@huawei.com (E.W.)

**Keywords:** mobile edge computing, immersive media, split rendering, Lyapunov optimization, service placement, online control

## Abstract

Immersive media services increasingly rely on edge-cloud rendering to deliver interactive visual content under dynamic network, computing, and mobility conditions. Rendering an entire scene as a single service couples interaction-sensitive foreground content with context-oriented background content, making it difficult to jointly control latency, quality, synchronization, and migration overhead. This paper studies sense-driven edge-cloud foreground–background split rendering for immersive media services. We formulate an online decision problem in which foreground and background rendering layers can be independently controlled under long-term system and migration cost budgets. The formulation turns structural scene separation into a coupled layer-state control problem by preserving asymmetric QoE roles and a common composition requirement. We propose SEELE, a Lyapunov-guided online control algorithm that represents accumulated budget pressure with two virtual queues and converts the long-term constrained problem into lightweight per-slot decisions. The resulting per-slot rule balances immediate QoE loss against queue-weighted system and migration costs. Under sustained resource and network stress, SEELE provides steady-state QoE statistically comparable to a pretrained PPO policy while significantly reducing synchronization violations and improving composition stability. It also improves steady-state QoE and system debt over deterministic and QoE-prioritized baselines. A prototype implementation and controlled characterization further validate split-stream deployment, runtime observability, practical control hooks, and the latency–capacity tradeoff of layered rendering.

## 1. Introduction

Immersive media services are becoming an important interface for entertainment, collaboration, training, and remote presence. Cloud gaming, extended reality (XR), telepresence, holographic communication, and interactive 3D streaming can provide more natural and responsive user experiences, but they require visual content to be rendered, transmitted, and updated according to user input within tight motion-to-photon and QoE budgets [1,2,3]. This latency requirement has pushed rendering architectures from remote cloud data centers toward nearby edge sites, and in 5G/MEC deployments the compute node may be placed close to the radio access network or even at the base-station level [2,4,5]. Such proximity reduces interaction delay and terminal workload, which is essential for lightweight immersive devices.

Bringing rendering closer to the radio edge, however, makes latency and capacity difficult to optimize at the same time. Near-edge and base-station-level resources provide short network paths, but they have limited compute and bandwidth capacity compared with regional edge or cloud clusters. Their operating state is also highly time-varying: wireless signal quality, available bandwidth, radio load, and access-point association change as users move and as neighboring sessions arrive. Cloud resources offer more rendering capacity, yet the longer access path can violate interaction latency. Serving the whole scene as a single rendered service therefore becomes inefficient in this extreme-edge regime. It may consume scarce edge compute for background content that is less interaction-sensitive, or it may move the entire service to the cloud and lose the latency benefit that motivated edge rendering in the first place.

Existing systems address parts of this conflict through prediction, remote rendering, service placement, migration, and collaborative rendering. Cloud gaming and edge/cloud VR systems show that prediction, offloading, and WebRTC-based rendering can reduce perceived delay or terminal-side workload [6,7,8]. Layered and foreground–background rendering further suggest that an immersive scene does not need to be controlled as a monolithic object. For example, recent wireless interactive VR work models foreground–background separation in an edge-device collaborative computing framework [9]. These approaches suggest keeping interaction-sensitive components close while assigning contextual components different rendering, quality, and placement choices. They leave open how these controls, synchronization repair, and migration should be coordinated under shared composition and recurring resource budgets.

However, foreground–background separation also creates a new online control problem. The foreground layer, such as user-manipulated objects, avatars, tools, and interaction-sensitive visual responses, has a strong dependence on latency and readiness. The background layer contributes more to visual continuity, scene detail, and immersion. The two layers must still be composed into a coherent output, so synchronization skew can hurt the user experience even when each individual layer is available, as also observed in multi-source immersive media delivery [10]. MEC placement and migration studies have addressed delay-reconfiguration tradeoffs for mobile services [11,12,13], but they usually operate on a single service object. Quality changes, synchronization repair, and migration therefore have layer-dependent effects in split rendering. A full-scene MEC placement model, which usually treats the service as one deployable object with one quality level and one delay measurement, cannot express this structure directly. The controller must decide when to improve QoE, when to conserve constrained edge resources, and when to avoid migration churn under uncertain future network, load, and mobility conditions.

This paper proposes SEELE, a sense-driven online control algorithm for edge-cloud foreground–background split rendering in immersive media services. Sense-driven control uses observed network, node, layered-service, and session conditions to form each online decision. Established Lyapunov drift-plus-penalty optimization provides long-term constraint handling [14]. SEELE contributes the layer-aware service state, composed QoE coupling, and finite-action cost structure that apply this foundation to split rendering. Two virtual queues represent system and migration debt, converting the long-term constrained problem into repeated per-slot decisions. The algorithm evaluates finite, interpretable actions for quality adaptation, synchronization repair, and deployment changes, becoming more cost-aware as debt accumulates. We evaluate SEELE with a simulation environment for split rendering sessions, heterogeneous terminal, edge, and cloud nodes, dynamic network and compute stress, and multiple baselines. Under sustained stress, SEELE provides steady-state QoE comparable to pretrained PPO while improving synchronization behavior, and it outperforms static placement and QoE-First control in steady-state QoE and long-term system debt. Ablation studies show that removing the system queue, migration queue, or synchronization action affects distinct aspects of the intended control behavior. A prototype implementation and controlled deployment characterization further validate split-stream composition, runtime observability, practical control hooks, and the operating tradeoff between interaction delay and rendering capacity.

The main contributions are summarized as follows:

- We formulate a sense-driven edge-cloud foreground–background split rendering model that makes structural scene separation a controllable layer state, capturing asymmetric QoE roles, common composition, and layer-aware placement, quality, synchronization, and migration controls.
- We design SEELE, a finite-action Lyapunov online control algorithm tailored to this layered state, with shared long-term queues for system and migration costs. We derive queue stability for the ideal joint DPP reference and implement SEELE as a practical sequential, queue-coupled approximation.
- We evaluate SEELE through simulation under mixed and stress conditions, and use a prototype implementation with controlled deployment profiles to validate split-stream composition, runtime observability, practical control hooks, and system-level latency–capacity tradeoffs.

The remainder of this paper is organized as follows. Section 2 reviews related work on edge/cloud immersive media, service placement and migration, and Lyapunov online control. Section 3 presents the system model and formulates the long-term constrained control problem. Section 4 introduces the SEELE algorithm and analyzes its queue stability. Section 5 reports the simulation and prototype evaluation. Section 6 concludes the paper.

## 2. Related Work

### 2.1. Edge and Cloud Immersive Media

XR streaming and mobile augmented reality have been widely studied as representative latency-sensitive immersive media services. Recent surveys show that XR delivery must jointly address high-rate visual traffic, low interaction latency, device energy limits, and user-perceived QoE [1,2,3]. Edge computing is therefore used to move rendering, perception, or media-processing workloads from the terminal to nearby compute nodes. This direction reduces terminal-side resource pressure, while making the final experience dependent on both wireless/network state and edge compute availability.

Recent systems optimize remote rendering and immersive media delivery from different angles. ZGaming uses image prediction to reduce perceived delay in 3D cloud gaming [6]. Edge/cloud VR experiments based on Unity Render Streaming quantify the latency and quality tradeoffs of interactive remote rendering over wireless networks [7]. Edge rendering architectures for multiuser XR further emphasize end-to-end latency and synchronization measurement in practical Web-based pipelines [8]. Work on wireless multi-user interactive VR has also introduced foreground–background separation as part of an edge-device collaborative computing framework [9]. In parallel, holographic communication studies show that synchronization across multiple media sources is itself a first-order quality issue [10].

Taken together, these studies show that immersive media needs edge-assisted rendering, runtime measurement, and synchronization-aware delivery. They also reveal a control gap. Many systems optimize a full stream, a full scene, or a specific rendering pipeline, while foreground–background split rendering requires the scheduler to reason about two service components with different QoE roles and a shared composition constraint. This paper treats synchronization and layer-specific quality adaptation as online service-control variables that directly affect scheduling decisions and system cost.

### 2.2. Service Placement and Migration in Mobile Edge Computing

Service placement and migration are central problems in mobile edge computing. Edge service placement decides where application components should run under latency, resource, and operating-cost constraints. For data-intensive edge applications, joint service placement and request scheduling can substantially affect both performance and resource efficiency [15]. For mobile users, the placement decision becomes dynamic because a service instance may need to follow the user across access regions. Ouyang et al. model this mobility-aware dynamic placement problem by jointly considering service delay and migration cost [11].

Recent work continues to study this delay-migration tradeoff under richer system models. Joint service migration and resource allocation in multi-cell MEC considers the coupling between migration, radio resources, and computing resources [16]. MoDEMS optimizes edge migration decisions under user mobility and demonstrates the practical importance of migration timing [12]. BigMEC studies scalable migration for mobile edge systems with many services and users [13]. Related studies on wide-area computing-network scheduling and IoT-centric fog placement further show that runtime task allocation, data movement, and decentralized service placement must be considered together in distributed edge environments [17,18]. Distributed dynamic placement in pervasive edge computing further shows how long-term service placement can be decomposed into online decisions while maintaining queue stability [19].

These placement and migration models provide useful foundations for reasoning about mobility, delay, and reconfiguration cost. In foreground–background split rendering, however, the controlled object is no longer a single service instance. Moving the foreground layer primarily affects interaction latency and interruption risk, adjusting the background quality primarily affects visual context and resource demand, and repairing inter-layer synchronization affects the composed output even when both individual streams remain available. The placement problem therefore becomes a layer-aware service-control problem with synchronization and quality adaptation in the same decision loop.

### 2.3. Lyapunov Optimization for Online Control

Lyapunov drift-plus-penalty optimization provides a standard way to transform long-term constrained stochastic optimization into online per-slot decisions [14]. The method introduces virtual queues for long-term constraints and then minimizes a one-slot drift-plus-penalty bound under future uncertainty. This structure is well suited to edge systems because the controller must often balance instantaneous service quality against accumulated resource pressure.

Recent MEC work has combined Lyapunov methods with online computation offloading, deep reinforcement learning, and dynamic service placement. LyDROO uses Lyapunov-guided reinforcement learning to stabilize online computation offloading under long-term constraints [20]. Distributed dynamic service placement in pervasive edge networks uses Lyapunov optimization to decompose a long-term placement objective into online subproblems and proves queue stability [19]. These studies show the value of virtual-queue reasoning for systems with uncertain future workloads.

Existing Lyapunov-based MEC formulations generally define controls at the task or service-instance level. Split rendering instead exposes a layered state with asymmetric QoE effects and a shared composition requirement. SEELE therefore instantiates established DPP machinery with layer-aware QoE and costs, a finite-action score for quality, synchronization, and migration control, and separate system and migration queues.

## 3. System Model and Problem Formulation

We consider an edge-cloud split-rendering service for multiple interactive immersive-media sessions. Each scene is divided into an interaction-sensitive foreground layer and a context-oriented background layer. The two layers can use different rendering nodes and quality levels, but they must be temporally aligned and combined before presentation. The online controller therefore determines their placement and quality, repairs inter-layer synchronization when needed, and migrates the layered service state as operating conditions change. This section defines the service state, QoE loss, cost processes, and resulting long-term constrained optimization problem.

**Figure 1:**
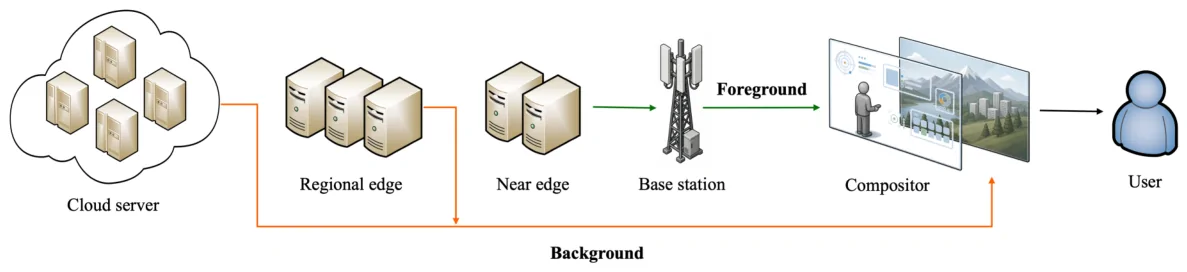
Representative edge-cloud architecture for foreground–background split rendering. The green path denotes the latency-sensitive foreground stream, and the orange path denotes the background stream supplied by cloud or regional-edge resources. The compositor is located at the user-side terminal, where it aligns and combines the two streams before delivering the composed scene to the user. This terminal also belongs to the candidate node set and may execute full-scene rendering or either rendering layer.

Figure 1 illustrates a representative deployment of the split-rendering pipeline. In the depicted configuration, cloud and regional-edge resources provide the background stream over a more elastic compute path, while a near-edge node provides the latency-sensitive foreground stream through the radio access path. The compositor at the user-side terminal receives both streams, aligns their presentation times, overlays the foreground content on the background scene, and sends one composed view to the user. In addition to composition, the terminal is a candidate rendering node in *N* and can execute full-scene rendering or either layer when selected. The depicted paths represent one feasible remote-rendering configuration; the rendering node and quality of each layer remain control variables, allowing the service to adapt its placement while preserving the common composition requirement.

### 3.1. Sessions, Nodes, and Split Rendering

The split-rendering service is described by active sessions, candidate nodes, and two rendering layers. Time is divided into slots t=0,1,2,…, and U(t) denotes the set of active sessions at slot *t*. The candidate rendering nodes are denoted by *N* and may include terminal-side resources, near-edge servers, regional edge servers, and cloud servers.

Each session is represented by a foreground–background layer set. Let

(1) L={fg,bg},

where fg denotes the foreground interaction layer and bg denotes the background context layer. The two layers can be controlled separately, but they are consumed jointly after composition, so the model must keep both their individual states and their coupled output.

The split model targets services that can expose interaction objects and contextual content as separate render passes while maintaining shared scene state. Representative examples include avatars, user-manipulated objects, tools, interface elements, and relatively stable environmental context. Strong cross-layer effects, including dynamic shadows, occlusion, illumination, and tightly coupled physics, may require auxiliary depth, alpha, or shared-state channels at the compositor. These extensions fit the same layer-state abstraction, while the current prototype focuses on foreground–background stream composition and synchronized input delivery.

The service state records the persistent variables changed by the controller. For session u∈U(t) and layer index l∈L, let $n_{u,l}\left(t\right)\in N$ be the rendering node and $q_{u,l}\left(t\right)\in Q$ be the selected quality level, where *Q* is a finite ordered quality set. Let $b_{u}\left(t\right)\geq 0$ denote the synchronization buffer and let $h_{u}\left(t\right)$ denote the finite service-transition state, including an ongoing migration interruption or recovery period. The controlled state of session *u* is

(2) $$x_{u}\left(t\right)=\left(n_{u,\mathrm{fg}}\left(t\right),n_{u,\mathrm{bg}}\left(t\right),q_{u,\mathrm{fg}}\left(t\right),q_{u,\mathrm{bg}}\left(t\right),b_{u}\left(t\right),h_{u}\left(t\right)\right).$$

The placement and quality entries specify the rendering configuration of both layers, while $b_{u}\left(t\right)$ and $h_{u}\left(t\right)$ retain the effects of synchronization and reconfiguration actions across slots. Lower-level encoder and transport choices are reflected through the selected quality level and the cost model defined below.

### 3.2. Runtime State and QoE Variables

The runtime state contains the exogenous and endogenous conditions that affect the next control decision. At the beginning of each slot, the controller observes a service-level state

(3) $$s\left(t\right)=\left(s_{\mathrm{network}}\left(t\right),s_{\mathrm{node}}\left(t\right),s_{\mathrm{service}}\left(t\right),s_{\mathrm{session}}\left(t\right)\right),$$

where $s_{\mathrm{network}}\left(t\right)$ describes network conditions, $s_{\mathrm{node}}\left(t\right)$ describes resource pressure at candidate nodes, $s_{\mathrm{service}}\left(t\right)$ describes the current placement and layer state, and $s_{\mathrm{session}}\left(t\right)$ describes session demand and mobility-related conditions.

The QoE variables are transient outputs derived from the controlled state and current runtime conditions. Let $d_{u}\left(t\right)$ denote foreground interaction latency, $\sigma_{u}\left(t\right)$ denote foreground–background synchronization skew, $g_{u}\left(t\right)$ denote the composed visual quality score, $f_{u}\left(t\right)$ denote effective frame rate, and $\eta_{u}\left(t\right)\in \left[0,1\right]$ denote service readiness. Readiness is computed from the transition state $h_{u}\left(t\right)$ together with node availability, load, and network loss; it is therefore an observed output rather than a directly controlled state variable. The corresponding requirements are the latency budget $D_{u}$, synchronization tolerance $S_{u}$, minimum composed-quality target $G_{u}$, and target frame rate $F_{u}$.

Synchronization is the explicit coupling term between the two rendering layers. Let $\tau_{u,\mathrm{fg}}\left(t\right)$ and $\tau_{u,\mathrm{bg}}\left(t\right)$ denote the foreground and background presentation times predicted at the composition endpoint before residual skew compensation. The synchronization buffer absorbs part of their difference, giving

(4) $$\sigma_{u}\left(t\right)={\left[\left|\tau_{u,\mathrm{fg}}\left(t\right)-\tau_{u,\mathrm{bg}}\left(t\right)\right|-b_{u}\left(t\right)\right]}^{+}.$$

Thus, $\sigma_{u}\left(t\right)$ is the residual foreground–background timing mismatch, measured in milliseconds, after the active synchronization buffer is applied. It is evaluated from the two presentation-time estimates in the current control slot; it is neither a moving average nor a maximum over multiple slots. The composed output can degrade even when both layers are individually available because residual mismatch breaks the consistency perceived by the user.

### 3.3. Control Actions and State Evolution

SEELE uses a small semantic action set to represent the main runtime interventions in split rendering. For each active session, the candidate actions are

(5) A={keep,degrade,upgrade,sync,migrate}.

The five actions have direct service meanings: keep preserves the current state, degrade lowers rendering or transmission demand, upgrade improves the selected quality level, sync repairs foreground–background alignment, and migrate changes deployment. The set *A* is the global action vocabulary, while $A_{u}\left(t\right)\subseteq A$ denotes the subset that is feasible for session *u* at slot *t*. For example, an already-low-quality session may not admit another degrade action, and a session without a feasible target node may not admit migrate. The selected action is denoted by $a_{u}\left(t\right)\in A_{u}\left(t\right)$, and

(6) $$a\left(t\right)=\{a_{u}\left(t\right):u\in U\left(t\right)\}$$

collects all per-session decisions made in the slot. This notation separates the fixed action design from the state-dependent feasibility of each decision.

The selected action updates the service state through a layer-level transition. We write this transition as

(7) $$x_{u}\left(t+1\right)=\Psi_{u}\left(x_{u}\left(t\right),a_{u}\left(t\right),s\left(t\right)\right),$$

where $\Psi_{u}\left(\cdot \right)$ captures how the selected action changes placement, quality, synchronization buffer, or service-transition state. The transient QoE variables, including readiness, are then recomputed from $x_{u}\left(t+1\right)$ and the observed runtime state.

Table 1 summarizes the main notation used in the system model and algorithm derivation.

### 3.4. QoE Loss and Cost Processes

The objective separates user-side degradation from provider-side operating pressure. Define the normalized violations  

(8) $$\begin{array}{cccc} e_{d,u}\left(t\right) & =\frac{{\left[d_{u}\left(t\right)-D_{u}\right]}^{+}}{D_{u}}, & e_{\sigma ,u}\left(t\right) & =\frac{{\left[\sigma_{u}\left(t\right)-S_{u}\right]}^{+}}{S_{u}},\\ e_{g,u}\left(t\right) & ={\left[G_{u}-g_{u}\left(t\right)\right]}^{+}, & e_{f,u}\left(t\right) & =\frac{{\left[F_{u}-f_{u}\left(t\right)\right]}^{+}}{F_{u}},\\ e_{\eta ,u}\left(t\right) & =I\{\eta_{u}\left(t\right)<\eta_{u}^{\min}\}, & & \end{array}$$

where ${\left[x\right]}^{+}=\max\left\{x,0\right\}$ and I{·} is the indicator function. For session *u*, the normalized QoE loss is

(9) $$J_{u}\left(t\right)=\frac{1}{C_{u}}\left(w_{d}e_{d,u}\left(t\right)+w_{\sigma }e_{\sigma ,u}\left(t\right)+w_{g}e_{g,u}\left(t\right)+w_{f}e_{f,u}\left(t\right)+w_{\eta }e_{\eta ,u}\left(t\right)+p_{u}\left(t\right)\right),$$

where $p_{u}\left(t\right)\geq 0$ is the transient penalty caused by migration interruption, migration recovery, or a quality switch, and $C_{u}>0$ is a normalization constant. The reported session-level utility is

(10) $$Q_{u}\left(t\right)=Q_{\max}{\left[1-J_{u}\left(t\right)\right]}^{+},$$

which is bounded on $\left[0,Q_{\max}\right]$. The aggregate slot loss optimized by the controller is

(11) $$J\left(t\right)=\frac{1}{\left|U(t)\right|}\sum_{u\in U(t)}J_{u}\left(t\right),$$

with J(t)=0 when no session is active.

The long-term constraints use explicit normalized system and migration cost processes. Let $\chi_{u,l}$ and $\beta_{u,l}$ denote the base compute and bandwidth demands of layer *l*, and let $s_{q}^{c}$, $s_{q}^{b}$, and $r_{q}$ denote the compute scale, bandwidth scale, and bitrate of quality level *q*. The resource demands under the selected quality are

(12) $$\begin{array}{cccc} r_{u,l}^{\mathrm{gpu}}\left(t\right) & =\chi_{u,l}s_{q_{u,l}\left(t\right)}^{c}, & r_{u,l}^{\mathrm{cpu}}\left(t\right) & =\alpha_{\mathrm{cpu}}r_{u,l}^{\mathrm{gpu}}\left(t\right),\\ r_{u,l}^{\mathrm{mem}}\left(t\right) & =\alpha_{\mathrm{mem}}r_{u,l}^{\mathrm{gpu}}\left(t\right), & r_{u,l}^{\mathrm{net}}\left(t\right) & =\beta_{u,l}s_{q_{u,l}\left(t\right)}^{b}+r_{q_{u,l}\left(t\right)}, \end{array}$$

where the four *r* terms represent GPU, CPU, video-memory, and bandwidth demand, respectively, and $\alpha_{\mathrm{cpu}}$ and $\alpha_{\mathrm{mem}}$ map GPU demand to the associated CPU and video-memory demand. Let M={gpu,cpu,mem,net} denote the resource types, $\omega_{m}$ their nonnegative weights, and ${\bar{r}}_{m}>0$ their reference scales. The normalized resource term is

(13) $$R_{u}\left(t\right)=\sum_{l\in L}\sum_{m\in M}\omega_{m}\frac{r_{u,l}^{m}\left(t\right)}{{\bar{r}}_{m}}.$$

Let $\rho_{u}\left(t\right)$ be the larger GPU-utilization ratio of the two nodes serving session *u*, let $\rho_{0}$ be the pressure threshold, and let $k(a_{u}\left(t\right))$ be the action cost. The per-session system cost is

(14) $$c_{\mathrm{sys},u}\left(t\right)=R_{u}\left(t\right)+\lambda_{1}{\left[\rho_{u}\left(t\right)-\rho_{0}\right]}^{+}+\lambda_{2}{\left[\rho_{u}\left(t\right)-1\right]}^{+2}+\lambda_{a}k\left(a_{u}\left(t\right)\right),$$

where $\lambda_{1}$, $\lambda_{2}$, and $\lambda_{a}$ are nonnegative calibration coefficients. The first term captures normalized resource consumption, the next two terms penalize sustained pressure and overload, and the final term represents the operating pressure of the selected intervention. Migration cost is a normalized event count:

(15) $$c_{\mathrm{mig},u}\left(t\right)=I\left\{a_{u}\left(t\right)=\mathrm{migrate}\;\mathrm{and}\;\mathrm{the}\;\mathrm{migration}\;\mathrm{is}\;\mathrm{executed}\right\}.$$

An executed migration contributes one event to $c_{\mathrm{mig},u}\left(t\right)$. Its interruption and recovery effects enter the transition state $h_{u}\left(t\right)$ and transient QoE penalty $p_{u}\left(t\right)$, while the intervention pressure enters the action term in Equation (14). The aggregate costs used in the virtual-queue updates are the active-session means

(16) $$c_{i}\left(t\right)=\frac{1}{\left|U(t)\right|}\sum_{u\in U(t)}c_{i,u}\left(t\right),\;i\in \left\{\mathrm{sys},\mathrm{mig}\right\},$$

with zero cost when no session is active. These definitions distinguish the optimized user-side loss from resource pressure and reconfiguration frequency while matching the quantities accumulated by the simulator.

The reference scales in Equation (13) make the resulting costs dimensionless, but the costs are not clipped to [0,1]. Their finite bounds follow from the assumed bounded demand, quality, action, and node-utilization ranges.

### 3.5. Problem Formulation

The online control problem minimizes long-term QoE loss under long-term cost budgets. The objective is

(17) $$\min_{\left\{a(t)\right\}}\underset{T\to \infty }{lim sup}\frac{1}{T}\sum_{t=0}^{T-1}E\left[J(t)\right],$$

subject to the system-cost budget

(18) $$\underset{T\to \infty }{lim sup}\frac{1}{T}\sum_{t=0}^{T-1}E\left[c_{\mathrm{sys}}\left(t\right)\right]\leq {\bar{c}}_{\mathrm{sys}},$$

the migration-cost budget

(19) $$\underset{T\to \infty }{lim sup}\frac{1}{T}\sum_{t=0}^{T-1}E\left[c_{\mathrm{mig}}\left(t\right)\right]\leq {\bar{c}}_{\mathrm{mig}},$$

and the per-session action feasibility constraint

(20) $$a_{u}\left(t\right)\in A_{u}\left(t\right),\;\forall u\in U\left(t\right),\;t\geq 0.$$

This formulation captures the central tradeoff in split rendering: improving the composed user experience may consume more edge-cloud resources or trigger more frequent reconfiguration. Section 4 develops SEELE to convert this long-term constrained problem into queue-aware per-slot decisions.

## 4. SEELE: Lyapunov-Guided Online Control

SEELE converts the long-term budget constraints into per-slot finite-action decisions. The method tracks accumulated cost pressure with virtual queues, derives a one-slot Lyapunov drift bound, and then adds QoE loss to obtain a drift-plus-penalty score. The resulting scheduler keeps the action space small while allowing the system-cost and migration-cost budgets to influence every decision.

### 4.1. Virtual Queues for Long-Term Cost Budgets

Virtual queues transform long-term budget violations into state variables. The constraints in Equations (18) and (19) cannot be checked from a single slot, so SEELE introduces one queue for each budget:

(21) $$Z_{\mathrm{sys}}\left(t+1\right)=\max\left\{Z_{\mathrm{sys}}\left(t\right)+c_{\mathrm{sys}}\left(t\right)-{\bar{c}}_{\mathrm{sys}},0\right\},$$

(22) $$Z_{\mathrm{mig}}\left(t+1\right)=\max\left\{Z_{\mathrm{mig}}\left(t\right)+c_{\mathrm{mig}}\left(t\right)-{\bar{c}}_{\mathrm{mig}},0\right\}.$$

The queue $Z_{\mathrm{sys}}\left(t\right)$ represents accumulated system-cost debt, and $Z_{\mathrm{mig}}\left(t\right)$ represents accumulated migration-cost debt. If the realized cost exceeds the corresponding budget in a slot, the queue increases by the excess; if the realized cost stays below the budget, the queue decreases by the corresponding shortfall unless the nonnegative projection sets it to zero. The queue values therefore summarize how much pressure previous decisions have placed on each long-term constraint.

The two queues are maintained separately because system pressure and migration pressure have different effects on split rendering. System pressure reflects sustained compute, bandwidth, and overload conditions, whereas migration pressure reflects reconfiguration churn and transient interruption. The dual-queue state allows the same action to be evaluated differently when the bottleneck is resource consumption and when the bottleneck is excessive migration.

### 4.2. Lyapunov Function and Drift Upper Bound

The next step is to convert the queue updates into a form that can guide a one-slot decision. A quadratic Lyapunov potential first measures the current amount of accumulated budget debt:

(23) $$\Phi \left(t\right)=\frac{1}{2}\left(Z_{\mathrm{sys}}^{2}\left(t\right)+Z_{\mathrm{mig}}^{2}\left(t\right)\right).$$

The conditional one-slot drift measures how the debt potential changes after the next decision:

(24) $$\Delta \left(t\right)=E\left[\Phi (t+1)-\Phi (t)|Z(t)\right],$$

where $Z\left(t\right)=\left(Z_{\mathrm{sys}}\left(t\right),Z_{\mathrm{mig}}\left(t\right)\right)$. The notation E[·∣Z(t)] denotes conditional expectation given the current virtual-queue state; the current debt values are treated as known, while the randomness of the next slot is averaged. The role of the drift is to make the long-term budgets visible at one slot. If an action tends to increase the queues, it also tends to increase the Lyapunov potential. Directly optimizing the next Lyapunov value is inconvenient because the queue update contains the projection operator in Equations (21) and (22). The following lemma is therefore used as a technical bridge: it removes the projection and exposes the action-dependent terms that later become the queue-weighted cost terms in the SEELE score.

**Lemma** **1** (Drift upper bound)**.** *Assume that $c_{\mathrm{sys}}\left(t\right)-{\bar{c}}_{\mathrm{sys}}$ and $c_{\mathrm{mig}}\left(t\right)-{\bar{c}}_{\mathrm{mig}}$ are bounded for all t. Then there exists a finite constant B such that*

(25) $$\Delta \left(t\right)\leq B+Z_{\mathrm{sys}}\left(t\right)E\left[c_{\mathrm{sys}}\left(t\right)-{\bar{c}}_{\mathrm{sys}}|Z\left(t\right)\right]+Z_{\mathrm{mig}}\left(t\right)E\left[c_{\mathrm{mig}}\left(t\right)-{\bar{c}}_{\mathrm{mig}}|Z\left(t\right)\right].$$

**Proof.**  The proof follows by squaring the projected queue update. Let

(26) $$y_{\mathrm{sys}}\left(t\right)=c_{\mathrm{sys}}\left(t\right)-{\bar{c}}_{\mathrm{sys}},\;y_{\mathrm{mig}}\left(t\right)=c_{\mathrm{mig}}\left(t\right)-{\bar{c}}_{\mathrm{mig}}.$$

Here $y_{i}\left(t\right)$ is the one-slot budget excess of queue *i*: it is positive when the corresponding cost exceeds its budget and negative when the slot stays below budget. For either queue i∈{sys,mig}, the update has the form $Z_{i}\left(t+1\right)=\max\left\{Z_{i}\left(t\right)+y_{i}\left(t\right),0\right\}$. Hence,

(27) $${\left(\max\{Z_{i}\left(t\right)+y_{i}\left(t\right),0\}\right)}^{2}\leq Z_{i}^{2}\left(t\right)+y_{i}^{2}\left(t\right)+2Z_{i}\left(t\right)y_{i}\left(t\right).$$

This inequality removes the projection operator without a case split; it is the standard step that converts the queue update into a quadratic drift bound. Subtracting $Z_{i}^{2}\left(t\right)$, summing the result over the two queues, and multiplying by 1/2 gives

(28) $$\Phi \left(t+1\right)-\Phi \left(t\right)\leq \frac{1}{2}\left(y_{\mathrm{sys}}^{2}\left(t\right)+y_{\mathrm{mig}}^{2}\left(t\right)\right)+Z_{\mathrm{sys}}\left(t\right)y_{\mathrm{sys}}\left(t\right)+Z_{\mathrm{mig}}\left(t\right)y_{\mathrm{mig}}\left(t\right).$$

Since the cost increments are bounded, their squared terms can be collected into a finite constant

(29) $$B=\frac{1}{2}\left({\left(y_{\mathrm{sys}}^{\max}\right)}^{2}+{\left(y_{\mathrm{mig}}^{\max}\right)}^{2}\right).$$

The constant *B* captures the largest possible one-slot quadratic change caused by bounded costs. It does not depend on the current queue values or on the particular candidate action. More explicitly, let $R_{\max}$ be Equation (13) evaluated at the configured maximum layer demands and quality scales, and let $\rho_{\max}$ be the maximum configured node-utilization ratio. A valid per-session bound is

(30) $$c_{\mathrm{sys}}^{\max}=R_{\max}+\lambda_{1}{\left[\rho_{\max}-\rho_{0}\right]}^{+}+\lambda_{2}{\left[\rho_{\max}-1\right]}^{+2}+\lambda_{a}k_{\max},\;c_{\mathrm{mig}}^{\max}=1.$$

Because the slot costs are active-session means, the same maxima bound their aggregate values. Therefore, one may set $y_{i}^{\max}=\max\left\{{\bar{c}}_{i},c_{i}^{\max}-{\bar{c}}_{i}\right\}$ in Equation (25). The finite quality and action sets, together with the configured demand and load extrema in Table 2 and Supplementary File S1, make these quantities finite. They are used only in the analysis and are not required by the online score. Taking conditional expectation given Z(t) gives Equation (25).    □

Lemma 1 is the point at which the long-term constraints become usable for online control. The constant *B* and the budget constants do not distinguish among candidate actions in a slot. The remaining action-dependent terms are the predicted system and migration costs multiplied by the current virtual queues. This observation leads directly to the drift-plus-penalty rule in the next subsection and is also used in the stability proof later in this section.

### 4.3. Drift-Plus-Penalty Decision Rule

The drift-plus-penalty rule trades immediate QoE loss against long-term cost debt. Using Lemma 1, SEELE adds a weighted QoE loss term to the drift bound:

(31) $$\Delta \left(t\right)+VE\left[J(t)|Z(t)\right],$$

where V>0 controls the emphasis on immediate QoE loss. A larger *V* makes SEELE more responsive to user-facing quality, while a smaller *V* gives stronger priority to queue stabilization.

SEELE performs per-session action selection with shared queue coupling. Exact joint minimization would require enumerating the Cartesian product of all per-session action sets, which is unsuitable for interactive control. Each active session therefore generates candidate actions from its current service state. Sessions are processed sequentially within a slot, so later decisions observe the node-load state produced by earlier accepted decisions. Migration candidates whose target nodes lack residual capacity are removed during feasibility checking. The remaining actions are coupled primarily through queue-weighted resource-pressure costs evaluated against the updated node-load state. SEELE therefore provides a tractable online approximation and does not claim to solve the global joint combinatorial problem.

The per-session score is obtained by keeping the terms in the DPP upper bound that depend on the candidate action. For a candidate action $a\in A_{u}\left(t\right)$ of session *u*, let ${\hat{J}}_{u}\left(t,a\right)$, ${\hat{c}}_{\mathrm{sys},u}\left(t,a\right)$, and ${\hat{c}}_{\mathrm{mig},u}\left(t,a\right)$ denote the predicted QoE loss, system cost contribution, and migration cost contribution under the current runtime state. The finite-action rule is

(32) $$a_{u}^{*}\left(t\right)=\arg\min_{a\in A_{u}\left(t\right)}\left[V{\hat{J}}_{u}\left(t,a\right)+\kappa_{\mathrm{sys}}Z_{\mathrm{sys}}\left(t\right){\hat{c}}_{\mathrm{sys},u}\left(t,a\right)+\kappa_{\mathrm{mig}}Z_{\mathrm{mig}}\left(t\right){\hat{c}}_{\mathrm{mig},u}\left(t,a\right)\right].$$

This score gives a direct interpretation of SEELE’s control behavior. The first term favors actions that reduce immediate QoE loss. The second term discourages actions that increase system cost when the system-cost queue is large. The third term discourages migration when the migration-cost queue is large. The positive factors $\kappa_{\mathrm{sys}}$ and $\kappa_{\mathrm{mig}}$ calibrate the numerical scales of the two normalized costs. The selected session-level actions form the joint action a(t) applied in the slot. When costs are additive across sessions, this rule matches the separable part of the one-slot DPP objective; when residual resources create coupling, it provides a tractable online approximation evaluated against the current shared system state.

The predicted terms in Equation (32) are obtained through model-based candidate evaluation. For each candidate action, SEELE constructs the candidate placement, quality, synchronization-buffer, and transition state; recomputes Equations (8)–(10) using the current network and node-load observations; and evaluates Equations (12)–(15). After the per-session actions are applied, their realized contributions are averaged according to Equation (16) before the two virtual queues are updated. The resource, quality, and score-calibration coefficients can be obtained through offline profiling and updated with runtime telemetry, without requiring a learned predictor.

### 4.4. Action Semantics

Each finite action represents a practical intervention on the layered service state. The action keep preserves the current placement, quality, and synchronization state, providing a reference candidate for the current operating point. The action degrade reduces rendering or transmission demand, which can lower system cost at the expense of visual quality. The action upgrade increases the selected quality level when residual resources and queue pressure permit. The action sync targets foreground–background alignment without requiring a placement change and is included because skew can dominate the composed experience even when both streams remain available. The action migrate changes the layer-aware deployment state when a target node can reduce latency or improve feasibility under mobility and load changes.

The semantic action set keeps the online scheduler lightweight while preserving the main control modes. Each primitive is evaluated through the same predicted QoE loss, system cost, and migration cost terms in Equation (32). More detailed encoder, buffering, or placement parameters can be handled inside the predictor associated with a semantic action, so the scheduler remains compact while reflecting the practical controls exposed by rendering and streaming systems.

### 4.5. Queue Stability and Constraint Satisfaction

Queue stability links the DPP objective back to the original long-term constraints. To state the guarantee precisely, consider an ideal joint DPP controller that minimizes the action-dependent upper bound over the complete feasible joint action set in each slot. This ideal controller provides the theoretical reference for the sequential implementation described in Section 4.3. The proof has two parts. First, it compares the ideal joint decision with a feasible benchmark policy that has budget slack and shows negative drift when the queues become large. Second, it uses the queue update itself to translate queue stability into long-term budget satisfaction.

**Theorem** **1** (Stability of the ideal joint DPP controller)**.** *Assume that per-slot costs and QoE loss are bounded, i.e., $0\leq J\left(t\right)\leq J_{\max}$. Suppose there exists a stationary randomized policy π that satisfies the two cost budgets with slack ϵ>0:*

(33) $$E\left[c_{\mathrm{sys}}^{\pi }\left(t\right)|Z\left(t\right)\right]\leq {\bar{c}}_{\mathrm{sys}}-\epsilon ,\;E\left[c_{\mathrm{mig}}^{\pi }\left(t\right)|Z\left(t\right)\right]\leq {\bar{c}}_{\mathrm{mig}}-\epsilon .$$

*Suppose the ideal joint DPP controller minimizes the action-dependent DPP upper bound over the feasible joint action set in every slot. Then its virtual queues are strongly stable:*

(34) $$\underset{T\to \infty }{lim sup}\frac{1}{T}\sum_{t=0}^{T-1}E\left[Z_{\mathrm{sys}}\left(t\right)+Z_{\mathrm{mig}}\left(t\right)\right]<\infty .$$

*Consequently, they are mean-rate stable, and the long-term constraints in Equations *(18)* and *(19)* are satisfied*.

**Proof.**  The proof first shows negative drift when the queues are large. Exact joint minimization makes the controller’s action-dependent bound no larger than the bound obtained from policy π. Combining Lemma 1 with Equation (33) gives

(35) $$\Delta \left(t\right)+VE\left[J^{\mathrm{joint}}\left(t\right)|Z\left(t\right)\right]\leq B+VE\left[J^{\pi }\left(t\right)|Z\left(t\right)\right]-\epsilon \left(Z_{\mathrm{sys}}\left(t\right)+Z_{\mathrm{mig}}\left(t\right)\right).$$

The slack ϵ is important: it means the benchmark policy leaves a margin below both budgets, so the queue-weighted terms become negative. When the queues are large, this negative term dominates the bounded QoE term and pulls the Lyapunov potential downward. Since the QoE loss is nonnegative and bounded by $J_{\max}$,

(36) $$\Delta \left(t\right)\leq B+VJ_{\max}-\epsilon \left(Z_{\mathrm{sys}}\left(t\right)+Z_{\mathrm{mig}}\left(t\right)\right).$$

Equation (36) is the key stability inequality. It says that the expected drift is upper-bounded by a constant minus a term proportional to the total queue backlog. Therefore, once the debt queues are sufficiently large, the expected drift is negative. Summing the drift bound converts this one-slot result into a time-average backlog bound. Taking expectation, summing over t=0,…,T−1, and telescoping the Lyapunov terms yields

(37) $$\frac{1}{T}\sum_{t=0}^{T-1}E\left[Z_{\mathrm{sys}}\left(t\right)+Z_{\mathrm{mig}}\left(t\right)\right]\leq \frac{B+VJ_{\max}}{\epsilon }+\frac{E[\Phi (0)]}{\epsilon T}.$$

Taking T→∞ proves strong stability because the time-average expected queue backlog is bounded by a finite constant.

The final step converts queue stability into budget satisfaction. For i∈{sys,mig}, let $c_{i}\left(t\right)$ denote the corresponding cost and let ${\bar{c}}_{i}$ denote its long-term budget. From the virtual queue update, $Z_{i}\left(t+1\right)\geq Z_{i}\left(t\right)+c_{i}\left(t\right)-{\bar{c}}_{i}$. Summing over time gives

(38) $$\sum_{t=0}^{T-1}c_{i}\left(t\right)\leq T{\bar{c}}_{i}+Z_{i}\left(T\right)-Z_{i}\left(0\right).$$

Dividing by *T*, taking expectation, and using mean-rate stability of $Z_{i}\left(T\right)$ gives the corresponding long-term average cost constraint. Intuitively, if the average excess cost stayed positive, the virtual queue would grow linearly; mean-rate stability rules out that linear growth. This proves the theorem.    □

The same proof accommodates a controller whose per-slot DPP value is within a queue-independent additive constant *C* of the joint minimum: *B* is replaced by B+C in Equations (35)–(37). Establishing such a uniform approximation bound for the sequential multi-session rule requires additional structure on cross-session resource coupling and is outside the present analysis. Accordingly, Theorem 1 is a guarantee for the ideal joint DPP reference. For the implemented sequential SEELE controller, the queue updates retain an exact implication: whenever its queues are mean-rate stable, the long-term budgets are satisfied by the final step of the proof. The long-horizon evaluation later in Section 5 provides empirical evidence of this behavior under the evaluated workload.

The analysis also gives the two queues an operational role in the implemented algorithm. When a queue grows, the corresponding cost term receives a larger weight in Equation (32); when the queue drains, SEELE can place more emphasis on immediate QoE repair. This mechanism turns long-term cost budgets into per-slot action preferences.

### 4.6. Online Algorithm and Complexity

SEELE is implemented as a lightweight per-slot scheduler. Algorithm 1 initializes the two virtual queues at zero. At each decision slot, it observes the runtime state and queue state, constructs feasible per-session action sets, evaluates the finite-action DPP score using shared queues and resource feasibility, applies the resulting joint action, and updates the queues using the realized costs.

**Algorithm** **1** SEELE Online Control Algorithm1: **Initialization:** Set $Z_{\mathrm{sys}}\left(0\right)=0$, $Z_{\mathrm{mig}}\left(0\right)=0$, and choose V>0.2: **for** each decision slot t=0,1,2,… **do**3:   Observe the runtime state s(t) and queue state Z(t).4:   Generate the feasible action set $A_{u}\left(t\right)$ for each active session u∈U(t).5:   Estimate ${\hat{J}}_{u}\left(t,a\right)$, ${\hat{c}}_{\mathrm{sys},u}\left(t,a\right)$, and ${\hat{c}}_{\mathrm{mig},u}\left(t,a\right)$ for each feasible session-action candidate.6:   Select $a_{u}^{*}\left(t\right)$ for each active session by minimizing Equation (32) under the current residual-resource state.7:   Apply the joint action $a\left(t\right)=\{a_{u}^{*}\left(t\right):u\in U\left(t\right)\}$ and observe $c_{\mathrm{sys}}\left(t\right)$ and $c_{\mathrm{mig}}\left(t\right)$.8:   Update $Z_{\mathrm{sys}}\left(t+1\right)$ and $Z_{\mathrm{mig}}\left(t+1\right)$ using Equations (21) and (22).9: **end for**


The complexity follows from semantic-action scoring and migration-target search. Let $C_{\mathrm{pred}}$ and $C_{\mathrm{feas}}$ denote the costs of one-step state/cost prediction and one placement-feasibility check, respectively. For each session, the scheduler scores at most $|A_{u}\left(t\right)|\leq 5$ semantic actions. The migration gate additionally searches the ordered foreground–background node pairs, filters them by reachability and residual capacity, and retains the target with the lowest predicted loss. The resulting per-slot complexity is

(39) $$O\;\left(\sum_{u\in U(t)}\left[|A_{u}\left(t\right)|C_{\mathrm{pred}}+{\left|N\right|}^{2}\left(C_{\mathrm{feas}}+C_{\mathrm{pred}}\right)\right]\right).$$

With the fixed two-layer representation, both checks operate on a constant number of resource and QoE terms. Hence the implementation is linear in |U(t)| for a fixed candidate topology and quadratic in |N| when the node set grows. The experiments use |N|=8 and a maximum of five semantic actions per session. An exhaustive joint-action solver would scale combinatorially across sessions and is not used in SEELE.

## 5. Experiments and Evaluation

### 5.1. Experimental Setup

We evaluate SEELE with a configuration-driven discrete-time simulator that implements the control loop in Section 3. One control slot represents 1 s. At each slot, the simulator updates access-path and node conditions, completes departing sessions, admits new sessions, and then invokes the selected policy for all active sessions. Policies compared within a scenario receive the same seeded session trace and disturbance schedule, which isolates the effect of the control rule.

The topology contains eight candidate nodes: one terminal, three near-edge nodes, two regional-edge nodes, and two cloud nodes. Their capacities and base path characteristics form the latency–capacity gradient summarized in Table 2. The terminal remains a member of the candidate set *N* and supports terminal-side full rendering or layer placement. Figure 1 focuses on a representative edge–cloud split path, while terminal-side rendering remains an alternative placement and full-rendering option. The relative capacity and latency ranges are anchored to Cloud VR provisioning studies, edge/cloud latency measurements, and cloud-gaming delay studies [21,22,23].

Session dynamics combine stochastic arrivals with heterogeneous demands. Inter-arrival times are exponentially distributed with a scenario-dependent mean of 3–4 slots, session durations are sampled uniformly from the configured interval, and compute and bandwidth demands receive independent bounded perturbations. Mobility enters the simulator at the service-control level through changes in the sensed access path: latency, bandwidth, jitter, loss, and availability vary over time and can change the relative suitability of candidate nodes. This metric-level abstraction covers observable Wi-Fi or cellular access variation without introducing a geometric trajectory predictor. Server contention is generated by tier-dependent background GPU load with two deterministic sinusoidal components, together with scheduled load bursts. All random draws use the scenario seed.

The evaluation instantiates the symbolic QoE model in Section 3 with $\eta_{u}^{\min}=0.90$, $C_{u}=100$, $Q_{\max}=100$, and $\left(w_{d},w_{\sigma },w_{g},w_{f},w_{\eta }\right)=\left(30,26,28,24,16\right)$. For the resource model, $\left(\alpha_{\mathrm{cpu}},\alpha_{\mathrm{mem}}\right)=\left(0.35,0.55\right)$, the resource weights $\left(\omega_{\mathrm{gpu}},\omega_{\mathrm{cpu}},\omega_{\mathrm{mem}},\omega_{\mathrm{net}}\right)$ are (0.45,0.20,0.20,0.15), and the corresponding reference scales $\left({\bar{r}}_{\mathrm{gpu}},{\bar{r}}_{\mathrm{cpu}},{\bar{r}}_{\mathrm{mem}},{\bar{r}}_{\mathrm{net}}\right)$ are (100,60,60,50). The pressure model uses $\rho_{0}=0.82$, $\left(\lambda_{1},\lambda_{2},\lambda_{a}\right)=\left(0.38,2.20,0.16\right)$, and action costs k(keep)=0, k(degrade)=0.8, k(upgrade)=2.8, k(sync)=0, and k(migrate)=18.

The transition calibration assigns an executed migration, a four-slot interruption, followed by a seven-slot recovery period. These stages add 58 and 16, respectively, to the unnormalized transient penalty $p_{u}\left(t\right)$, while a quality-level switch adds 1.2. Migration candidates use an eight-window transition evaluation to combine the initial interruption with the persistent placement effect. The finite-action score uses $\kappa_{\mathrm{sys}}=0.035$ and $\kappa_{\mathrm{mig}}=1$ in the main comparison. These values, together with scenario-specific overrides and profiling fields, are included in Supplementary File S1.

The session requirements and default control values have direct operational interpretations. The foreground latency budgets remain below the approximately 100 ms interaction target commonly used for latency-sensitive cloud gaming, while tighter profiles create deliberate stress [23]. The synchronization tolerances correspond approximately to one presentation-frame interval at 45–60 FPS; this application-level requirement reflects the importance of frame alignment in composed multi-source immersive media [10]. The system budget 0.65 limits the active-session mean of the normalized cost in Equation (16), and the migration budget 0.02 limits the executed-migration indicator to an average of 2% per active session-slot. We use V=5.0 as the QoE-responsive operating point at the upper end of the sensitivity range examined later in this section; lower values place progressively greater emphasis on queue pressure. In practice, the two budgets should first be set from the admissible resource and reconfiguration envelope, after which *V* can be swept to select the desired QoE–cost tradeoff. These defaults are fixed across the main comparisons.

The main comparative evaluation includes a sustained-stress setting and four targeted profiles that emphasize edge-capacity pressure, cloud-latency pressure, runtime bursts, and long-horizon debt accumulation. The sustained-stress setting combines stochastic session arrivals and departures, access-path variation, and overlapping external GPU-load events over a 540-slot horizon. The overall, stress-scenario, and ablation bar charts aggregate 30 independent seeds. Their error bars show two-sided 95% confidence intervals for the mean based on Student’s *t* distribution. Since all policies receive the same stochastic trace for a given seed, policy differences are assessed with two-sided paired *t*-tests; Holm correction is applied across the reported comparisons, and Cohen’s $d_{z}$ reports the paired effect size. Supplementary File S1 contains the simulator source, configuration files, fixed seed lists, unit tests, reproduction pipeline, and aggregate outputs used by the paper. Each run also stores its fully merged configuration, allowing the workload and disturbance sequence to be reconstructed without relying on undocumented defaults.

The learning-based comparison uses a parameter-sharing proximal policy optimization (PPO) agent [24]. Each per-session decision is one agent step, and the policy receives a 38-dimensional state containing the session’s current QoE and constraint residuals, placement and quality state, shared resource pressure, virtual-queue values, and recent action context. Absolute time and session-lifecycle progress are excluded. The agent selects from the same five semantic actions and uses the same candidate-feasibility and migration-target rules as SEELE; a synchronization repair is masked when no synchronization violation is observed. The actor and critic each use two 64-unit tanh hidden layers. Generalized advantages are computed separately for each session trajectory so that returns are not propagated between users. Training comprises 75 episodes and 588,685 decision steps drawn only from independent mixed-stress traces, with learning rate $3\times 10^{-4}$, discount factor 0.995, generalized-advantage parameter 0.95, and clipping ratio 0.2. Its reward is exactly the negative realized SEELE drift-plus-penalty score, with no additional reward terms. Training seeds 1001–1075 are disjoint from the 30 evaluation seeds. The resulting checkpoint is frozen during every reported comparison; hence, the main PPO results represent a pretrained deployment policy rather than online learning on the test traces. The sustained-stress setting and four targeted profiles are excluded from training and therefore test distribution-shift robustness.

The measurement-robustness test separates sensing error from the realized environment. For each positive latency, bandwidth, jitter, current-load, or external-load value *y* used by the controller, the sensed value is $\tilde{y}=y\left(1+e_{y}\right)$, where $e_{y}$ is independently sampled from U[−ρ,ρ] at each state refresh and ρ∈{0,0.05,0.10,0.20}. The true state continues to determine the service transition, realized QoE, and realized costs. Consequently, differences from the zero-error case measure sensitivity to imperfect state observation rather than a change in the underlying disturbance trace.

### 5.2. Baselines and Metrics

We compare SEELE with full-scene baselines, a structure-only split-rendering baseline, a QoE-prioritized online baseline, a learned PPO controller, and SEELE ablations. The compared policies are:

- **Edge-Full**: the full scene is served from edge-side resources without foreground–background separation.
- **Cloud-Full**: the full scene is served from cloud resources.
- **Static-Layered**: foreground and background layers are split, but the placement and control policy remain static.
- **QoE-First**: a threshold heuristic uses only the current measured state. It repairs a sufficiently large synchronization error, lowers quality under latency, frame-rate, or resource pressure, raises quality when resources are lightly loaded, and otherwise keeps the current state. It performs neither candidate-state optimization nor migration.
- **PPO**: a parameter-sharing actor–critic policy learns per-session action selection from the common state and finite-action interface. Its reward is the negative realized SEELE drift-plus-penalty expression, with no additional reward terms.
- **SEELE variants**: ablations remove the system-cost queue, migration-cost queue, or synchronization action.

**Figure 2:**
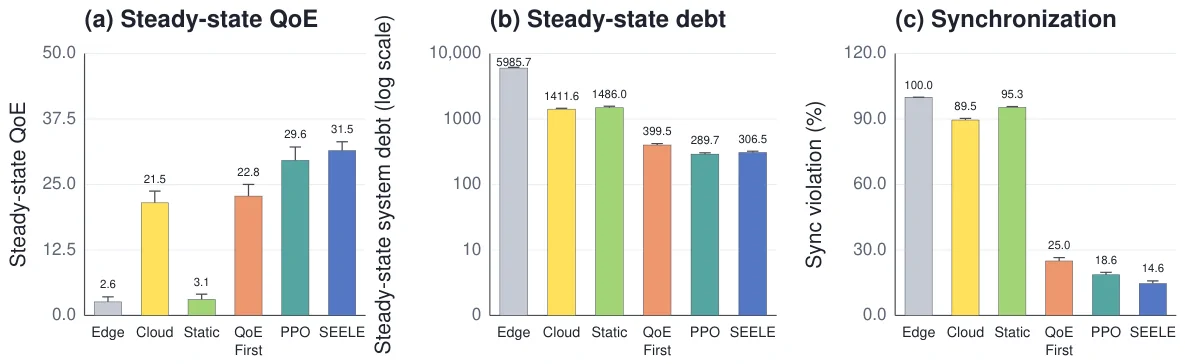
Steady-state performance under sustained resource and network stress. QoE and debt are averaged over the final third of each 540-slot run. Bars report means over 30 independent seeds, and error bars denote 95% confidence intervals.

**Figure 3:**
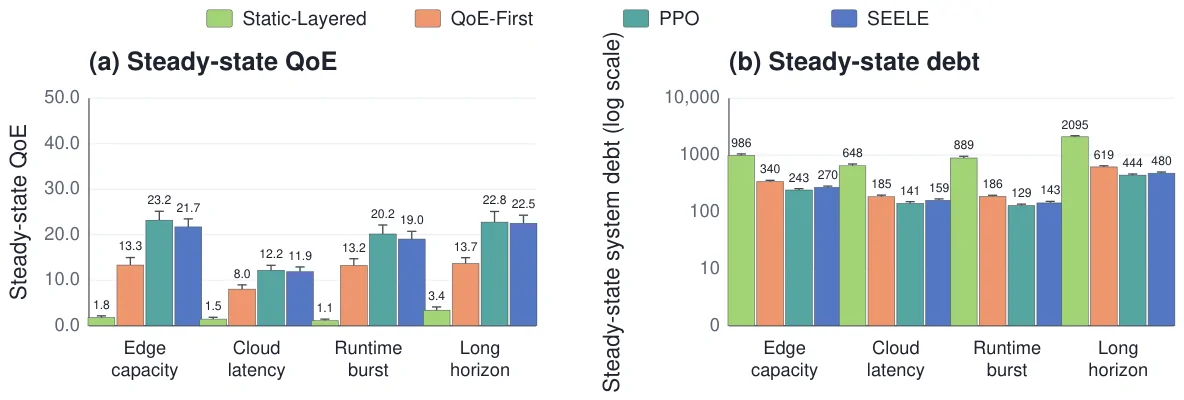
Steady-state performance under four stress conditions. QoE and debt use the final third of each run. Bars report means over 30 independent seeds, and error bars denote 95% confidence intervals.

The metrics cover sustained user experience, long-term constraint pressure, reconfiguration, synchronization, and runtime cost. To evaluate operation after resource pressure and virtual-queue dynamics have developed, let $T_{\mathrm{ss}}=\left\{\left\lceil 2T/3\right\rceil ,\ldots ,T-1\right\}$ denote the final third of a run. The primary utility metric is

(40) $${\bar{Q}}_{\mathrm{ss}}=\frac{\sum_{t\in T_{\mathrm{ss}}}\sum_{u\in U(t)}Q_{u}\left(t\right)}{\sum_{t\in T_{\mathrm{ss}}}\left|U\left(t\right)\right|},$$

which uses the same fixed time fraction for every policy, scenario, and seed. Steady-state system debt is the corresponding final-third mean of the virtual queue system. We also report migration count, synchronization-violation ratio, composition stability, late bad-window ratio, and decision time. QoE loss remains the per-slot term optimized by both SEELE and PPO. Figure 2 and Figure 3 use the same bounded [0,100] utility definition and the same final-third window. Because debt spans more than two orders of magnitude, panel (b) uses a clearly labeled $\log_{10}\left(1+x\right)$ axis while the labels above the bars show untransformed values.

### 5.3. Steady-State Performance

Figure 2 compares the six policies over 540 slots with 60 sessions. SEELE reaches a steady-state QoE of 31.46±1.69, while PPO reaches 29.62±2.54; the paired difference is not statistically significant after correction (p=0.094, $d_{z}=0.32$). Their steady-state debts are 306.46±15.02 and 289.73±13.64, respectively. The more important separation appears in composition behavior: SEELE reduces the synchronization-violation ratio from 18.64%±1.11% to 14.63%±1.19% (Holm-adjusted $p=2.85\times 10^{-7}$, $|d_{z}|=1.30$) and improves composition stability from 0.881±0.010 to 0.923±0.011 (Holm-adjusted $p=3.16\times 10^{-8}$, $d_{z}=1.48$). Thus, SEELE’s statistically supported advantage over PPO in this sustained setting is a more reliable foreground–background composition at comparable steady-state QoE.

The comparison also separates the benefits of service structure from those of online control. Edge-Full and Static-Layered collapse under sustained capacity pressure, while Cloud-Full retains 21.50±2.24 steady-state QoE at a debt above 1400. The simple QoE-First heuristic reaches 22.79±2.23 steady-state QoE and 399.53±20.74 steady-state debt, so it improves on fixed operation without approaching the two advanced controllers. SEELE exceeds QoE-First by 8.67 steady-state QoE points and reduces steady-state debt by 93.06 (both Holm-adjusted $p<2.4\times 10^{-11}$). These results support a balanced interpretation: PPO and SEELE provide comparable sustained utility, while SEELE offers significantly better synchronization behavior without offline policy training.

### 5.4. Stress Scenario Performance

Figure 3 evaluates robustness under four profiles excluded from PPO training. SEELE reaches steady-state QoE values of 21.72, 11.89, 19.05, and 22.51 under edge-capacity, cloud-latency, runtime-burst, and long-horizon stress, respectively. The corresponding QoE-First steady-state values are 13.34, 8.05, 13.25, and 13.67. SEELE also reduces steady-state debt from 340.07, 184.73, 185.90, and 618.92 under QoE-First to 270.01, 158.76, 143.21, and 480.35. All eight paired steady-state QoE and debt improvements remain significant after Holm correction ($p<5.6\times 10^{-8}$). The simple current-threshold heuristic therefore improves on static operation, but its lack of candidate-state and accumulated-queue evaluation limits sustained operation under combined resource and composition pressure.

PPO reaches steady-state QoE values of 23.20, 12.18, 20.16, and 22.77 in the same profiles. Its 1.47-point advantage under edge-capacity stress is significant after Holm correction, whereas its differences under cloud latency, runtime burst, and long horizon are not significant. PPO has lower steady-state debt in all four profiles. Table 3 shows the complementary composition behavior. Composition stability is the session-slot mean of $1-\min\{v_{u}^{\mathrm{sync}}\left(t\right),1\}$, where $v_{u}^{\mathrm{sync}}\left(t\right)$ is the normalized synchronization-threshold exceedance. It captures exceedance severity, whereas the synchronization-violation ratio records how often a positive exceedance occurs; larger stability values are better.

SEELE significantly improves composition stability and reduces synchronization violations under edge-capacity, cloud-latency, and runtime-burst stress. The cloud-latency reduction is the largest: synchronization violation falls by 11.92 percentage points and stability rises by 0.118. Under runtime bursts, SEELE also exhibits a 2.45-point smaller early-to-late QoE drop (Holm-adjusted $p=2.11\times 10^{-4}$), indicating faster sustained recovery. The long-horizon composition differences are smaller and do not remain significant after family-wise correction. Taken together, the profiles show that pretrained PPO generally maintains lower debt, while SEELE preserves comparable sustained utility in three cases and provides stronger foreground–background alignment under transient edge and network disturbances without offline policy training.

### 5.5. Runtime Adaptation Behavior

**Figure 4:**
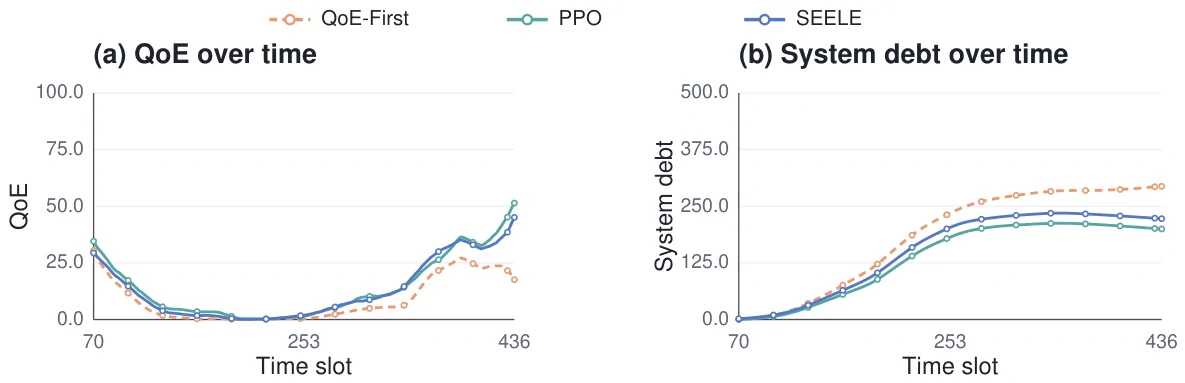
Runtime adaptation in the sustained-stress setting for representative seed 4. Panels (**a**,**b**) show 18-slot moving averages of per-session QoE and system debt.

**Figure 5:**
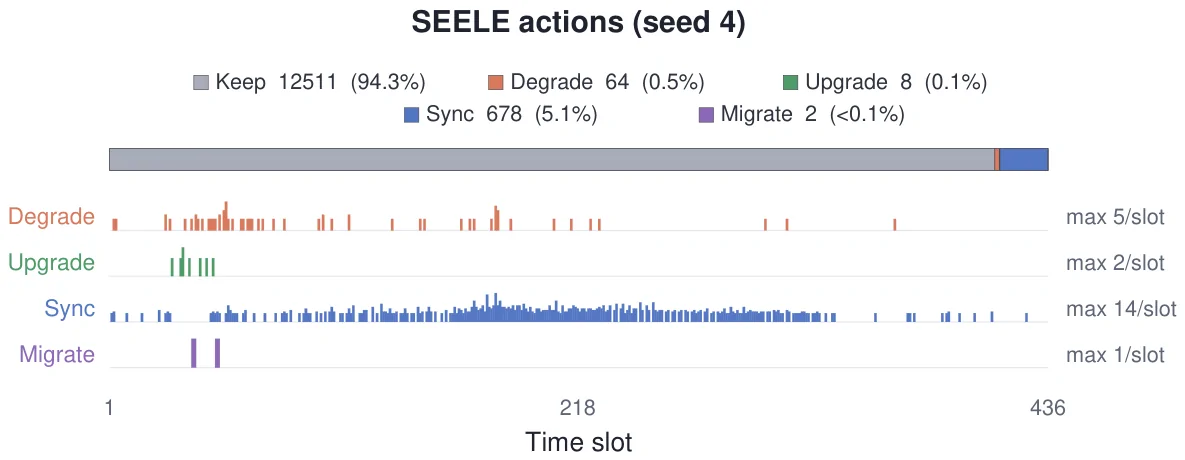
SEELE action composition and correction timeline for representative seed 4. The full control horizon is shown; bar height gives the number of active sessions selecting each non-keep action in a slot.

Figure 4 and Figure 5 connect the performance trajectory with the concrete actions selected by SEELE. Figure 4 includes PPO, SEELE, and QoE-First under the identical seed-4 disturbance trace. PPO and SEELE recover to similar QoE levels, while PPO retains somewhat lower debt and QoE-First accumulates the most debt. Synchronization is the principal SEELE correction during the disturbed portion of the trace; quality upgrades and degradations are used more selectively as quality and resource pressure change. Across the full control horizon, 94.3% of the 13,263 session-slot decisions are keep, 5.1% are synchronization repairs, and the two quality directions together account for 0.6%. The high keep share is expected because an action is recorded separately for every active session in every slot, whereas only sessions whose measured or predicted state crosses a control condition require a change. Two migrations occur at slots 40 and 51, where the predicted persistent placement gains (0.16 and 0.34) pass the minimum-gain and interruption-cost gate. Their sparse occurrence illustrates that migration is reserved for selected placement improvements rather than used as a routine response to every disturbance.

The runtime trace illustrates how queue feedback changes action preference over time. When the system-cost queue increases, actions that would add more pressure receive higher scores even if they are attractive for immediate QoE. When the queue is small, SEELE can upgrade quality or perform synchronization repair more freely. The sustained synchronization activity during the high-debt interval explains why SEELE can preserve composition alignment without spending every slot on a quality or placement change. The observed trajectory therefore matches the DPP rule derived in Section 4.

### 5.6. Mechanism Ablation

**Figure 6:**
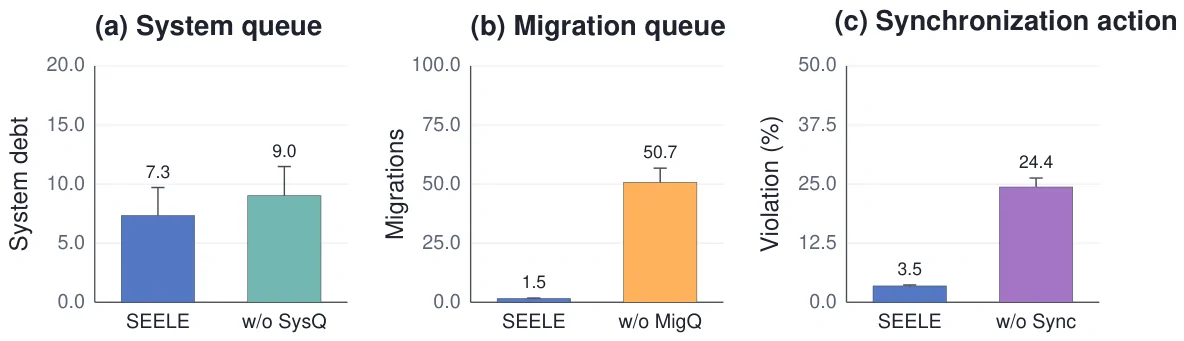
Targeted ablation of SEELE mechanisms. Panels (**a**,**c**) use the resource-and-synchronization stress setting; panel (**b**) uses the repeated-handoff migration setting. Bars report means over 30 independent seeds, and error bars denote 95% confidence intervals.

Figure 6 uses a targeted stress setting for each mechanism so that the corresponding control signal is active. Removing the system-cost queue increases system debt from 7.34 to 9.02 (Holm-adjusted $p=6.61\times 10^{-6}$, $|d_{z}|=1.15$), showing the effect of accumulated resource pressure. In the repeated-handoff setting, removing the migration-cost queue increases the migration count from 1.53 to 50.73 (Holm-adjusted $p=3.13\times 10^{-15}$, $|d_{z}|=3.05$) and migration debt from 1.22 to 8.21 (Holm-adjusted $p=7.82\times 10^{-12}$, $|d_{z}|=2.23$). Removing the synchronization action raises the synchronization-violation ratio from 3.46% to 24.38% (Holm-adjusted $p=1.15\times 10^{-18}$, $|d_{z}|=4.10$).

The targeted ablation also shows why utility should be interpreted together with synchronization and long-term cost metrics. Without the migration queue, the controller can reconfigure more aggressively and the QoE score rises from 34.93 to 36.81, but the mean interruption cost increases from 12.3 to 405.9. Removing synchronization repair instead lowers the QoE score from 31.55 to 25.51 while substantially increasing skew violations. The complete controller therefore favors a more balanced operating point rather than maximizing any single short-term metric.

### 5.7. Parameter Sensitivity, Measurement Robustness, and Overhead

**Figure 7:**
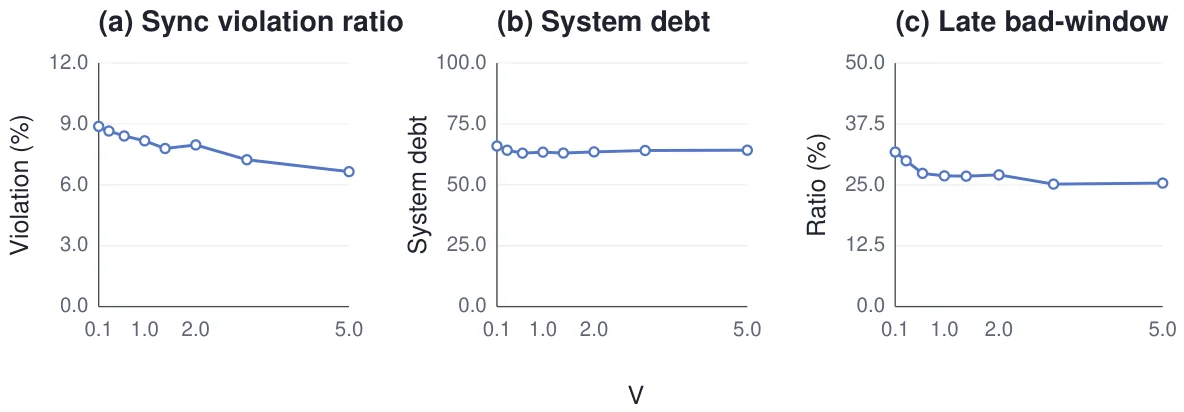
Sensitivity to the Lyapunov control parameter *V* in the dedicated sensitivity setting. Larger *V* emphasizes immediate QoE repair and synchronization, while smaller *V* makes the policy more conservative with long-term cost debt.

Figure 7 studies the Lyapunov parameter *V* in a dedicated long-horizon setting. As *V* increases from 0.1 to 5.0, the synchronization-violation ratio decreases from 8.9% to 6.6%, and the late bad-window ratio decreases from 31.7% to 25.4%. System debt remains in a comparatively narrow range of 63.0–66.0 because the queue term continues to regulate the same budget. The sweep therefore shows that larger *V* gives the immediate QoE and synchronization terms more influence, while the long-term cost response remains active.

Table 4 complements the *V* sweep with the two long-term budgets. Relaxing the system budget from 0.55 to 0.75 reduces system debt from 19.65 to 4.00 because each slot receives a larger cost allowance. The tighter budget lowers average system cost from 0.714 to 0.687 and permits only 0.30 migrations, whereas the relaxed budget permits 7.07. In the migration-active setting, relaxing the migration budget from 0.01 to 0.04 increases the migration count from 8.93 to 20.37 and raises the QoE score from 36.90 to 37.61, while migration debt decreases from 0.097 to 0.069 because the larger allowance absorbs more reconfiguration cost. These monotone control responses support setting each budget from the admissible resource or reconfiguration envelope and then selecting *V* for the desired QoE–cost tradeoff. The defaults 0.65 and 0.02 are the central values of the tested ranges and remain fixed across the main comparisons.

The observation-error results show that prediction error increases monotonically from 0.007 to 0.151 as ρ rises to 20%. Relative to zero injected error, the 20% case changes the QoE score by +5.1%, average system cost by −1.4%, and system debt by −12.9%, but the migration count rises from 3.97 to 52.20. Thus, the aggregate operating point remains bounded, while noisy observations trigger substantially more placement corrections at the largest error level. The non-monotonic QoE and debt values reflect the nonlinear action response to unbiased bounded perturbations rather than a claim that sensing error is beneficial. Moderate error up to 10% causes a smaller correction increase, whereas the 20% result identifies a practical reason to filter telemetry or add migration hysteresis in deployment.

**Figure 8:**
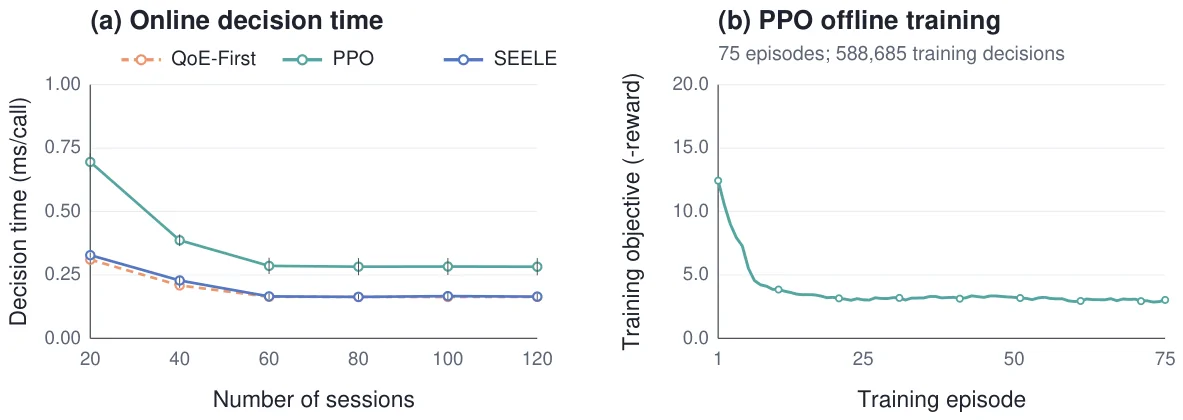
Online decision overhead and PPO training requirement. Panel (**a**) reports steady-state decision time, and panel (**b**) shows the five-episode moving average of the PPO training objective.

Figure 8a reports the per-decision overhead as the number of sessions increases. The timed region covers candidate construction, state preparation, and action selection, while simulation-state updates, file I/O, and plotting are excluded. The benchmark was executed with Python 3.12.1, PyTorch 2.2.2, and NumPy 1.26.4 on macOS 26.4.1 using a MacBook Pro (Apple Inc., Cupertino, CA, USA) with an 11-core Apple M3 Pro processor and 18 GB of unified memory. Across the tested loads, the maximum mean decision times are 0.310 ms for QoE-First, 0.695 ms for PPO, and 0.328 ms for SEELE. PPO is therefore approximately 2.1 times slower than SEELE at the maximum observed means, although all three remain below 1 ms per decision on this platform. A separate fresh-process measurement over ten launches, including Python and PyTorch initialization, checkpoint loading, and the first action, gives a median PPO startup time of 0.903 s. This is a deployment dependency rather than evidence that PPO cannot meet the per-slot deadline after initialization.

Figure 8b exposes the preparation hidden by a frozen-policy comparison. The PPO objective improves rapidly during its early training episodes and stabilizes only after repeated interaction, whereas the reported SEELE policy requires neither exploration nor a learned checkpoint. The final PPO numbers elsewhere in this section are measured only after all 75 episodes and 588,685 training decisions have completed. Under sustained test stress, the two controllers provide statistically comparable steady-state QoE; SEELE additionally provides immediate model-driven operation, lower steady-state computation, explicit constraint logic, and a controller path already exercised by the prototype.

### 5.8. Queue Convergence and Budget Satisfaction

**Figure 9:**
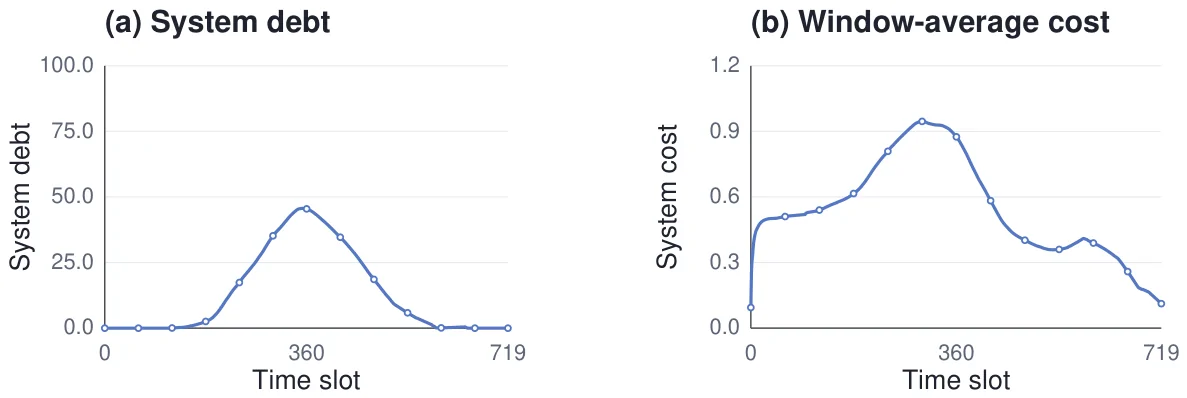
Queue and cost behavior over a 720-slot long-horizon experiment. Panel (**a**) reports system debt and panel (**b**) reports the 96-slot moving average of realized system cost, both averaged over five runs.

Figure 9 connects the empirical behavior to the stability argument in Section 4. The left panel reports the system-debt process represented by the virtual queue $Z_{\mathrm{sys}}\left(t\right)$, averaged over five runs. The queue increases when recent decisions exceed the budget and decreases when the realized cost stays below the budget. Over 720 slots, it remains bounded, reaches a peak mean of 46.98, and drains to zero by the end of the experiment.

The right panel reports a 96-slot moving average so that repeated periods above and below the budget are visible instead of being hidden by cumulative smoothing. The corresponding cumulative average, evaluated separately from the plotted windowed curve, reaches 0.521±0.023 at slot 720, below the system-cost budget of 0.650. Together, the bounded queue trajectory, repeated queue drainage, and final cumulative average provide empirical evidence that SEELE regulates sustained budget pressure in this experiment.

These trajectories are the empirical counterpart of the virtual-queue argument. Short-term violations are allowed when they help preserve QoE, but persistent violation is penalized by the queue, which shifts later decisions toward lower-cost actions. The important observation is that the queue provides feedback that prevents high-cost behavior from persisting over the long horizon.

### 5.9. Prototype Implementation and Controlled Characterization

We implemented a foreground–background split rendering prototype to examine the service abstraction used by SEELE under a deployable streaming stack. The prototype is built on Unreal Engine 5.4 Pixel Streaming and a browser-side WebGL compositor. The rendering side packages full-scene, foreground, and background services as independent components using Docker 29.1.1. Each component exposes an encoded WebRTC stream through its own signaling endpoint. In layered mode, the browser establishes separate foreground and background streams, removes the foreground color key, composites both video frames, and forwards user input events to the active rendering endpoints. The two endpoints can be co-located or assigned to different deployment nodes, corresponding to the layer-level placement state in Section 3.

**Figure 10:**
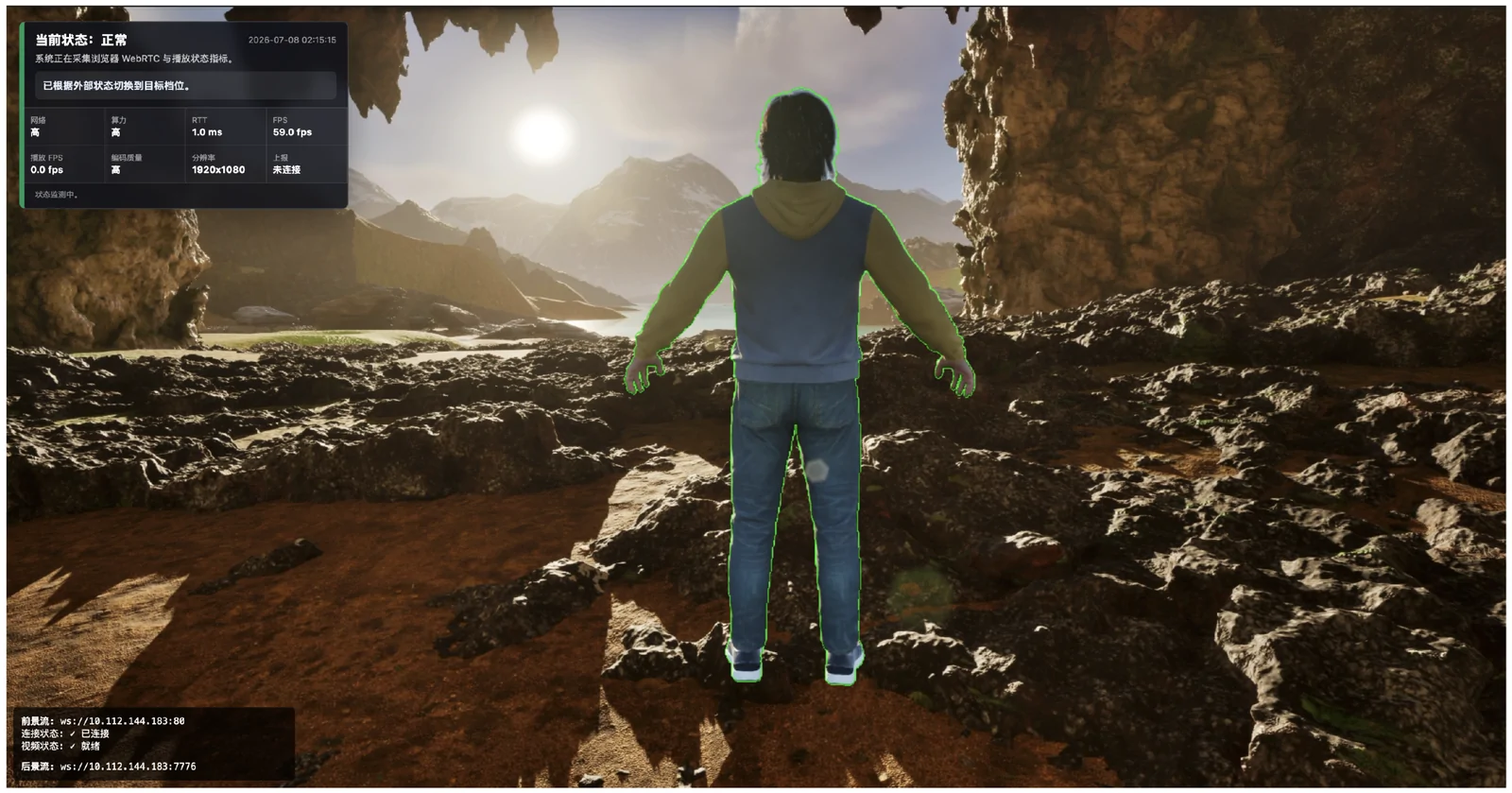
Prototype runtime snapshot. The browser compositor receives split foreground and background streams, overlays the interactive foreground object on the background scene, and displays runtime monitoring information. The Chinese interface labels report the current service status, network and compute levels, RTT, FPS, playback FPS, encoding quality, resolution, reporting status, and the connection and readiness states of the foreground and background streams.

The runtime interface exposes the state needed by SEELE-style online decisions. Browser-side instrumentation combines WebRTC statistics, playback state, and application-level stream status. It collects round-trip time, jitter, bitrate, frame rate, resolution, packet loss, decoded and dropped frames, stream readiness, candidate-pair state, and per-stream playback time. These measurements provide practical inputs for latency, quality, readiness, and synchronization-related state estimation. Figure 10 illustrates the prototype in layered rendering mode, where the foreground avatar is highlighted and the monitoring panel reports the current runtime state.

The prototype also exposes practical hooks for the finite actions. The actions degrade and upgrade map to bitrate, frame-rate, quantization, and resolution controls in the Pixel Streaming pipeline. Per-stream playback times, joint input delivery, and temporary input protection provide compositor-side hooks for synchronization monitoring and repair. Independently addressable containers and signaling endpoints provide the deployment hook required for foreground or background endpoint switching. These mechanisms ground the layer-level service state and show how runtime observations and semantic actions can be connected to a practical edge-cloud rendering platform.

The four controlled profiles in Table 5 characterize how compute location and rendering structure affect the prototype operating point. The runs use 1920 × 1080 video, a 45 s collection period, and a 10 s warm-up exclusion. An Ubuntu 24.04.3 LTS GPU server equipped with an NVIDIA GeForce RTX 3090 GPU (NVIDIA Corporation, Santa Clara, CA, USA) emulates resource tiers through GPU clock limits, CPU stress, and symmetric tc/netem delay, while Google Chrome 151.0.0.0 on a Mac client collects WebRTC and playback statistics. The remote-cloud, terminal, and single-edge profiles are direct full-scene measurements. The layered-edge profile combines independent clean measurements of a foreground service with 1.00 normalized compute and a background service with 1.07 normalized compute; its effective FPS is the slower measured stream. The interaction-latency proxy adds RTT, frame interval, per-frame WebRTC processing delay, and per-frame jitter-buffer delay. Remote-cloud rendering sustains 60 FPS but introduces a 51.03-ms RTT, while the compute-limited terminal profile reaches only 12.47 FPS despite its 1 ms RTT. The single-edge profile reaches 43.50 FPS and an 11.09 ms RTT. The layered profile reaches an effective 54.12 FPS and a 97.23 ms latency proxy, corresponding to a 24.4% FPS increase and a 31.3% proxy reduction relative to single-edge rendering. A separate simultaneous dual-stream run measures 59.11 FPS for the foreground and 59.03 FPS for the background, confirming that the composed update rate follows the slower stream. Its aggregate normalized compute is therefore 2.07, with an aggregate bitrate of 105.32 Mbps, compared with 1.00 and 91.72 Mbps for the single-edge profile. The measurements consequently expose the central latency–capacity tradeoff that motivates SEELE’s long-term cost regulation. Because the resource tiers are emulated and the layered profile aggregates independently collected clean runs, the table provides a controlled system characterization rather than a general performance distribution across heterogeneous deployments.

We further measured the operation of the compositor and quality-control hooks, as summarized in Table 6. For browser composition, the existing WebGL function was instrumented with performance.now() around each foreground–background composition call. Three measurement windows at 1920 × 1080 produced 6385 calls on Chrome 151 with an Apple M3 Pro client. The mean CPU-side call time was 0.219 ms and the per-window 95th percentile was at most 0.40 ms. This measurement covers browser-side WebGL command submission; transport, decoding, display scan-out, and motion-to-photon delay remain outside its scope. For control response, an archived runtime trace contains seven resolution-changing commands, comprising four degradations and three upgrades or recoveries. The requested resolution appeared in the first subsequent browser sample for every transition. With approximately one-second polling, the mean observed upper bound was 1.006 s and the maximum was 1.007 s.

The operational measurements quantify both the responsiveness and the resource cost of the split-stream path. The two quality directions use the same Pixel Streaming controls and are reflected in browser-observed resolution changes. The layered profile increases aggregate bitrate by 14.8% and normalized compute by 107.0% relative to the single-edge profile, while the effective FPS increases by 24.4%. The measured ratios include the second rendering service and stream and therefore represent the complete controlled rendering profiles. They complement the four-profile characterization by showing that composition and control are responsive at runtime while the additional transport and rendering capacity remains visible to the long-term cost controller.

Taken together, the prototype provides system-level support for the service abstraction used by SEELE. It demonstrates that foreground and background rendering can be independently deployed and monitored, that their streams can be composed into a unified view, and that the runtime stack exposes controls corresponding to the algorithm’s semantic actions. These capabilities provide a practical sensing-and-actuation foundation for integrating the online controller into a deployable edge-cloud rendering system.

## 6. Conclusions

This paper presented SEELE, a sense-driven online control algorithm for edge-cloud foreground–background split rendering in immersive media services. By separating the interaction-sensitive foreground layer from the context-oriented background layer, SEELE enables finer-grained control over placement, quality adaptation, synchronization, and migration. The algorithm formulates these decisions as a finite-action online control problem and uses two Lyapunov virtual queues to regulate long-term system and migration costs. The drift-plus-penalty derivation converts long-term budgets into per-slot queue-aware action scores, allowing SEELE to balance immediate QoE loss and accumulated cost debt. Under sustained resource and network stress, SEELE provides steady-state QoE statistically comparable to pretrained PPO, while significantly reducing synchronization violations and improving composition stability. It also improves steady-state QoE and system debt over deterministic full-scene, static, and QoE-prioritized baselines, without requiring offline policy training. Ablation studies confirm the distinct roles of the system-cost queue, migration-cost queue, and synchronization action. The prototype implementation demonstrates split-stream composition, runtime telemetry, and practical control hooks, while its controlled characterization illustrates how layered deployment trades additional distributed resources for improved frame rate and interaction-path latency. Future work will extend SEELE with trace-driven evaluation, explicit compositor-side skew control, and hybrid model-based and learned control for larger multi-user deployments.

## Figures and Tables

**Table 1:** Main notation used in the system model and SEELE derivation.

Symbol	Meaning
U(t)	Active session set at slot *t*
*N*	Candidate rendering node set
*L*	Rendering layer set, {fg,bg}
*l*	Rendering layer index, l∈L
*Q*	Ordered rendering quality level set
$x_{u}\left(t\right)$	Placement, quality, synchronization-buffer, and transition state of session *u*
$b_{u}\left(t\right),h_{u}\left(t\right)$	Synchronization buffer and finite interruption/recovery state of session *u*
s(t)	Observed runtime state at slot *t*
$A_{u}\left(t\right)$	Feasible action set for session *u* at slot *t*
$J_{u}\left(t\right),J\left(t\right)$	Per-session and aggregate QoE loss at slot *t*
$d_{u}\left(t\right),\sigma_{u}\left(t\right),g_{u}\left(t\right),f_{u}\left(t\right),\eta_{u}\left(t\right)$	Latency, synchronization skew, composed quality score, frame rate, and readiness variables
$c_{\mathrm{sys}}\left(t\right)$	System cost at slot *t*
$c_{\mathrm{mig}}\left(t\right)$	Migration cost at slot *t*
${\bar{c}}_{\mathrm{sys}},{\bar{c}}_{\mathrm{mig}}$	Long-term system and migration cost budgets
$Z_{\mathrm{sys}}\left(t\right),Z_{\mathrm{mig}}\left(t\right)$	Virtual queues for system and migration cost debt
*V*	Drift-plus-penalty parameter controlling QoE emphasis

**Table 2:** Key simulator parameters used in the evaluation. Detailed per-node and per-event values, fixed seeds, and merged run configurations are included in Supplementary File S1.

Category	Configured Values
Control clock	1 s per slot; identical seeded traces are used for policies compared within each scenario.
Node resources	Terminal: GPU/VRAM/CPU =54/40/45; near edge: GPU 62–115, VRAM 48–88, CPU 60–100; regional edge: GPU 170–260, VRAM 140–190, CPU 155–205; cloud: GPU 520–720, VRAM 390–540, CPU 430–600.
Base path conditions	Terminal, near-edge, regional-edge, and cloud latency: 3, 14–22, 28–35, and 62–78 ms; bandwidth: 180, 235–280, 360–390, and 520–680 Mbps, respectively. Base jitter is 1–10 ms and loss probability is 0.001–0.008.
Quality levels	Q1: 720p/30 FPS/4 Mbps; Q2: 900p/45 FPS/7 Mbps; Q3: 1080p/60 FPS/11 Mbps. Their compute scales are 0.65, 1.00, and 1.45.
Session process	Exponential inter-arrival mean: 3–4 slots; configured duration intervals span 90–300 slots; 56–70 sessions and 360–540 slots per main experiment. The convergence diagnostic uses 720 slots.
Layer demands	Foreground/background base compute: 16.8–19.0/50.0–64.0 units; base bandwidth: 7.0–8.2/14.2–17.0 units. Compute perturbation is ±18–22% and bandwidth perturbation is ±12.6–15.4%.
Session requirements	Foreground latency budget $D_{u}=74$–88 ms; synchronization tolerance $S_{u}=17$–22 ms; minimum composed-quality target 0.78–0.82.
Path variation	Scenario-wide latency scale 1.10–1.30, bandwidth scale 0.74–0.82, added jitter 4.0–5.6 ms, added loss 0.006–0.008, and uniform latency noise up to 4.5–5.5 ms. Scheduled node events apply additional latency, bandwidth, jitter, loss, and availability changes.
Background load	Base GPU-load ratios are 0.05–0.06 (terminal), 0.46–0.68 (near edge), 0.40–0.54 (regional edge), and 0.24–0.32 (cloud), with sinusoidal variation and scheduled load increments.
Default control values	V=5.0, ${\bar{c}}_{\mathrm{sys}}=0.65$, and ${\bar{c}}_{\mathrm{mig}}=0.02$.

**Table 3:** Steady-state utility and composition robustness of SEELE and PPO under stress profiles excluded from PPO training. Each pair is reported as SEELE/PPO. Upward and downward arrows indicate that higher and lower values are preferable, respectively.

Scenario	Steady-State QoE ↑	Composition Stability ↑	Sync Violation (%) ↓
Edge capacity	21.72/23.20	0.942/0.903	12.99/17.51
Cloud latency	11.89/12.18	0.910/0.793	15.57/27.49
Runtime burst	19.05/20.16	0.957/0.930	9.84/12.54
Long horizon	22.51/22.77	0.823/0.804	25.38/26.63

**Table 4:** Budget sensitivity and measurement robustness. System-budget and measurement-error rows use the mixed-stress setting, while migration-budget rows use a migration-active fast-handoff setting. Entries are means over 30 independent seeds, and the value after ± is the 95% confidence margin. Relevant debt denotes system debt or migration debt according to the varied constraint.

Factor	Setting	QoE Score	Relevant Debt	Migrations	Prediction Error
System budget	0.55	35.14±1.61	19.65±2.42	0.30±0.30	–
System budget	0.65	34.76±1.55	9.10±2.45	3.97±1.28	–
System budget	0.75	34.44±1.50	4.00±1.75	7.07±1.42	–
Migration budget	0.01	36.90±1.72	0.097±0.015	8.93±0.96	–
Migration budget	0.02	37.57±1.87	0.086±0.014	14.47±1.66	–
Migration budget	0.04	37.61±1.83	0.069±0.011	20.37±2.16	–
Measurement error	0%	34.76±1.55	9.10±2.45	3.97±1.28	0.007±0.001
Measurement error	5%	36.06±1.41	7.45±2.03	6.33±1.67	0.038±0.002
Measurement error	10%	36.67±1.48	7.50±2.33	13.10±3.34	0.074±0.004
Measurement error	20%	36.53±1.30	7.93±2.28	52.20±10.42	0.151±0.008

**Table 5:** Controlled prototype characterization at 1920 × 1080. Normalized compute is relative to the single-edge profile, and the latency proxy is derived from browser WebRTC statistics.

Metric	Remote Cloud	Terminal	Single Edge	Layered Edge
Normalized compute	4.48	0.26	1.00	1.00+1.07
Decoded FPS	60.00	12.47	43.50	54.12
Measured RTT (ms)	51.03	1.00	11.09	11.00
Latency proxy (ms)	143.00	204.04	141.45	97.23
Aggregate bitrate (Mbps)	34.49	56.75	91.72	105.32

**Table 6:** Prototype operational measurements. The quality-response value is bounded by the monitoring interval, and the stream overhead is an aggregate controlled-profile comparison.

Measurement	Observed Result	Evidence and Scope
WebGL composition call	0.219 ms mean; 0.40 ms P95	6385 calls in three 1080p measurement windows
Quality-profile response	1.006 s mean; 1.007 s maximum	Seven target-resolution transitions observed at approximately 1 s polling
Layered transport overhead	105.32 vs. 91.72 Mbps (+14.8%)	Aggregate bitrate relative to the single-edge profile
Layered compute overhead	2.07 vs. 1.00 (+107.0%)	Aggregate normalized compute relative to the single-edge profile

## Data Availability

The simulator source code, experiment configurations, fixed seeds, reproduction scripts, aggregate results, and prototype measurement traces are provided in Supplementary File S1.

## Supplementary Materials

The following supporting information can be downloaded at https://www.mdpi.com/article/10.3390/s26175561/s1, Supplementary File S1 contains the SEELE simulator source code, experiment configurations, fixed seed lists, unit tests, reproduction scripts, aggregate result files, and prototype measurement traces used in this study.

## References

[1] Wang H., Dong H., El Saddik A. (2025). Immersive multimedia communication: State-of-the-art on eXtended reality streaming. ACM Trans. Multimed. Comput. Commun. Appl..

[2] Siriwardhana Y., Porambage P., Liyanage M., Ylianttila M. (2021). A survey on mobile augmented reality with 5G mobile edge computing: Architectures, applications, and technical aspects. IEEE Commun. Surv. Tutor..

[3] Stafidas E., Foukalas F. (2024). A survey on enabling XR services in beyond 5G mobile networks. IEEE Access.

[4] Satyanarayanan M. (2017). The emergence of edge computing. Computer.

[5] Shi W., Cao J., Zhang Q., Li Y., Xu L. (2016). Edge computing: Vision and challenges. IEEE Internet Things J..

[6] Wu J., Guan Y., Mao Q., Cui Y., Guo Z., Zhang X. ZGaming: Zero-latency 3D cloud gaming by image prediction. Proceedings of the ACM SIGCOMM 2023 Conference.

[7] Casasnovas M., Michaelides C., Carrascosa-Zamacois M., Bellalta B. (2024). Experimental evaluation of interactive edge/cloud virtual reality gaming over Wi-Fi using Unity Render Streaming. Comput. Commun..

[8] Yeregui I., Mejías D., Pacho G., Viola R., Astorga J., Montagud M. Edge rendering architecture for multiuser XR experiences and E2E performance assessment. Proceedings of the 2024 IEEE International Symposium on Broadband Multimedia Systems and Broadcasting (BMSB).

[9] Xu C., Chen Z., Tao M., Zhang W. (2025). Wireless multi-user interactive virtual reality in metaverse with edge-device collaborative computing. IEEE Trans. Wirel. Commun..

[10] Anmulwar S., Wang N., Huynh V.S.H., Bryant S., Yang J., Tafazolli R. (2023). HoloSync: Frame synchronisation for multi-source holographic teleportation applications. IEEE Trans. Multimed..

[11] Ouyang T., Zhou Z., Chen X. (2018). Follow me at the edge: Mobility-aware dynamic service placement for mobile edge computing. IEEE J. Sel. Areas Commun..

[12] Kim T., Dhawaskar Sathyanarayana S., Chen S., Im Y., Zhang X., Ha S., Joe-Wong C. (2023). MoDEMS: Optimizing edge computing migrations for user mobility. IEEE J. Sel. Areas Commun..

[13] Brandherm F., Gedeon J., Abboud O., Mühlhäuser M. BigMEC: Scalable service migration for mobile edge computing. Proceedings of the 2022 IEEE/ACM 7th Symposium on Edge Computing (SEC).

[14] Neely M.J. (2010). Stochastic Network Optimization with Application to Communication and Queueing Systems.

[15] Farhadi V., Mehmeti F., He T., La Porta T.F., Khamfroush H., Wang S., Chan K.S., Poularakis K. (2021). Service placement and request scheduling for data-intensive applications in edge clouds. IEEE/ACM Trans. Netw..

[16] Liang Z., Liu Y., Lok T.M., Huang K. (2021). Multi-cell mobile edge computing: Joint service migration and resource allocation. IEEE Trans. Wirel. Commun..

[17] Song Y., Song P., Gao W., Liu S., Huo Z. (2025). Runtime collaborative scheduling of resources in computing power network oriented to the wide-area distributed intelligent computing environment. Big Data Res..

[18] Sulimani H., Sajjad A.M., Alghamdi W.Y., Kaiwartya O., Jan T., Simoff S., Prasad M. (2023). Reinforcement optimization for decentralized service placement policy in IoT-centric fog environment. Trans. Emerg. Telecommun. Technol..

[19] Ning Z., Dong P., Wang X., Wang S., Hu X., Guo S., Qiu T., Hu B., Kwok R.Y.K. (2021). Distributed and dynamic service placement in pervasive edge computing networks. IEEE Trans. Parallel Distrib. Syst..

[20] Bi S., Huang L., Wang H., Zhang Y.-J.A. (2021). Lyapunov-guided deep reinforcement learning for stable online computation offloading in mobile-edge computing networks. IEEE Trans. Wirel. Commun..

[21] Choi H.-J., Komuro N., Kim W.-S. (2024). Microservices-based resource provisioning for multi-user cloud VR in edge networks. Electronics.

[22] Ali-Eldin A., Wang B., Shenoy P. The hidden cost of the edge: A performance comparison of edge and cloud latencies. Proceedings of the International Conference for High Performance Computing, Networking, Storage and Analysis (SC21).

[23] Shea R., Liu J., Ngai E.C.-H., Cui Y. (2013). Cloud gaming: Architecture and performance. IEEE Netw..

[24] Schulman J., Wolski F., Dhariwal P., Radford A., Klimov O. (2017). Proximal policy optimization algorithms. arXiv.

